# Anti-bradycardia pacing—impact on patients with HFpEF: a systematic review

**DOI:** 10.1007/s10741-024-10382-1

**Published:** 2024-01-28

**Authors:** Alexandru Ababei, Luciana Andreea Hrib, Adalia Cristiana Iancu, Andra-Valeria Hadarag, Ahmad Khebbaiz, Radu Vătășescu, Ștefan Bogdan

**Affiliations:** 1 Carol Davila University of Medicine and Pharmacy, Bulevardul Eroii Sanitari 8, Sector 5, 050474 Bucharest, Romania; 2 Clinic Emergency Hospital, Bucharest, Romania; 3 Elias Emergency Hospital, Bucharest, Romania

**Keywords:** HFpEF, Left ventricular diastolic dysfunction, Cardiac pacing, Heart rate, Pacemaker

## Abstract

Heart failure with preserved ejection fraction (HFpEF) has become an emerging concern. The protective effect of bradycardia in patients with reduced ejection fraction using beta-blockers or ivabradine does not improve symptoms in HFpEF. This review aims to assess current data regarding the impact of anti-bradycardia pacing in patients with HFpEF. A search was conducted on PubMed, ScienceDirect, Springer, and Wiley Online Library, selecting studies from 2013 to 2023. Relevant and eligible prospective studies and randomized controlled trials were included. Functional status, quality of life, and echocardiographic parameters were assessed. Six studies conformed to the selection criteria. Four were prospective studies with a total of 90 patients analyzed. Two were randomized controlled trials with a total of 129 patients assessed. The 6-min walk test (6MWT) and the Minnesota Living with Heart Failure Questionnaire (MLHFQ) score improved in all prospective studies. My-PACE trial showed improvements in MLHFQ score (*p* < 0.001), significant relative lowering in NT-proBNP levels (*p* = 0.02), and an increased mean daily activity in the personalized accelerated pacing group compared to usual care. RAPID-HF trial proved that pacemaker implantation to enhance exercise heart rate (HR) did not improve exercise capacity and was associated with increased adverse events. HFpEF requires a more individualized approach and quality of life management. This review demonstrates that higher resting HR by atrial pacing may improve symptoms and even outcomes in HFpEF, while a higher adaptive rate during exertion has not been proven beneficial.

**Graphical Abstract:**

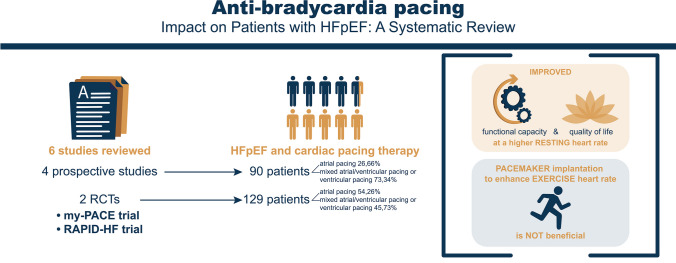

## Introduction

Heart failure with preserved ejection fraction (HFpEF) has become an emerging concern over the past 30 years, as it currently represents approximately half of all heart failure (HF) cases being associated with high morbidity and mortality. It is a syndrome characterized by the presence of HF symptoms and signs due to abnormal diastolic function in the setting of preserved left ventricular ejection fraction (LVEF) and the absence of significant valvular or ischemic heart disease [1]. 

The understanding of HFpEF has been complex due to its challenging diagnosis and underlying heterogeneous etiologies. Although difficult to prove, the increasing prevalence of HFpEF may be partly related to its increased recognition as a clinical entity. However, studies such as the Framingham Heart Study (FHS) and the Rochester Epidemiology Project have provided insights into HFpEF, showing that patients with HFpEF are predominantly elderly female and have a high prevalence of comorbidities such as hypertension, coronary artery disease (CAD), diabetes mellitus, obesity, anemia, chronic kidney disease, atrial fibrillation (AF), and chronic obstructive pulmonary disease [2]. 

The overall prevalence of HFpEF has been reported to be 1.1–5.5% in the general population. In addition, the prevalence of HFpEF increases with age, being much higher in individuals aged ≥ 80 years compared to those aged 25–49 years, as shown in studies such as Prevention of Renal and Vascular End-Stage Disease (PREVEND), FHS and Cardiovascular Health Study [3].

### HFpEF physiopathology

The relationship between left ventricular (LV) pressure and volume in a heart with diastolic dysfunction suggests a passive stiffness of the cardiac wall, revealed by an increase of diastolic pressure at any volume. The pressure–volume loop in this condition indicates an upward and leftward shift compared to a normal heart. Furthermore, in HFpEF, filling pressure is elevated, and wall thickness is relatively increased [4].

**Fig. 1:**
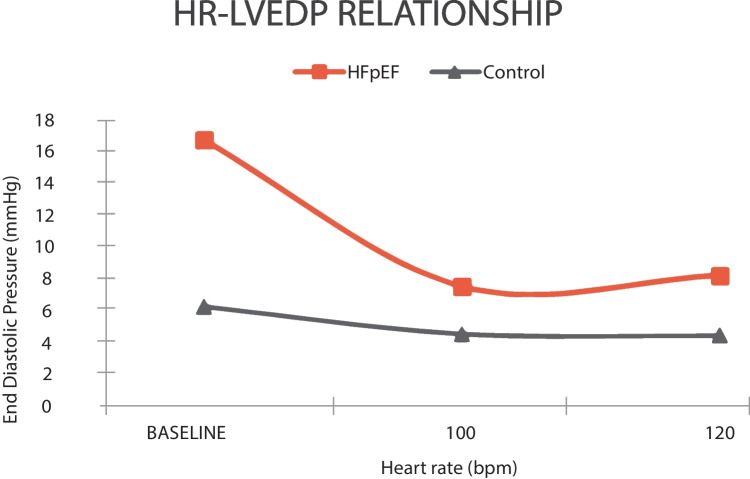
The relationship between HR and LVEDP at baseline (median: 66 bpm in HFpEF, 72 bpm in control), 100 bpm, and 120 bpm in patients with HFpEF and control [7]. Graphics program used: Microsoft Corporation. (2018). Microsoft Excel. Retrieved from https://office.microsoft.com/excel

A study supervised by Silverman et al. [5] highlighted a decrease of LV filling pressures in patients with and without HFpEF at a higher HR (125 bpm) compared to resting HR, while Nambiar et al. [6] documented a reduction of NT-proBNP. The heart rate–left ventricular end-diastolic pressure (HR–LVEDP) relationship has a different pattern in HFpEF patients versus control: there is an improvement of LVEDP on a higher HR compared to baseline, but further increase of HR determines an elevation of LVEDP, as shown in Fig. 1 [7]. Karliner et al. [8] explained the reduction of LVEDP by an accelerated myocardial contraction and relaxation due to increased activity of the calcium pump of the sarcoplasmic reticulum and cellular calcium handling reflected in the myocardial force–frequency and relaxation–frequency relationships [5, 7, 9–13]. These findings are in contrast to a study conducted by Borlaug et al. [14] about the abnormal increase of filling pressure during exercise, mainly because of an activation of the sympathetic tone with more significant changes in contractility–relaxation dynamics: the action of skeletal muscle pumps, which increases systemic venous return, and adjustments to peripheral resistance [5, 14–16].

Moreover, the relationship between a higher HR and an improvement of LVEDP is overridden by the tendency of reduction in blood pressure between 95 and 125 bpm in patients with HFpEF because of a more significant decrease in LV volumes compared to the control group, resulting in a blunting or inversion of the HR–cardiac output relationship [5]. Wachter et al. [7] showed that stroke volume decreased in HFpEF patients compared to a control group who maintained it during 100 and 120 bpm, subsequently outbalancing the increase in HR, leading to only a slight improvement in cardiac output at 100 bpm and no further increase at 120 bpm. At HRs > 170 bpm, the associated volume loss can be so profound that stroke volume and cardiac output approach zero. Therefore, patients with concentrically remodeled LVs are more prone to develop hypotension with tachycardia because of a morpho-pathological loss in volume due to the parallel addition of new myofibrils [5].

### HFpEF and bradycardia

HF has a prevalence of 10–20% among sick sinus syndrome (SSS) patients [17]. Moreover, the risk of HF and AF remains elevated in the SSS patients group compared to non-SSS individuals [18]. The first line of treatment for symptomatic patients with SSS is the implantation of a pacemaker [19].

Several studies have explored the relationship between HR and clinical outcomes in HF patients, showing that the protective effect of bradycardia does not apply to patients with very low HR. Still, also sinus bradycardia can be a facilitating factor in the appearance of overt HF. This hypothesis was supported by the THEOPACE trial [20], which showed that an increase in HR by DDD pacing or oral theophylline in patients with SSS and a baseline rate < 50 bpm reduced the incidence of overt HF [21]. Furthermore, the decreased HR effect caused by ivabradine did not improve clinical outcomes in patients with HFpEF [22].

Cleland et al. [23] conducted a meta-analysis assessing the use of beta-blockers in HF patients with either sinus rhythm or AF. The results indicate that in patients in sinus rhythm with LVEF < 40%, there is an improvement in LV systolic function accompanied by a reduction in cardiovascular morbidity and mortality. Similar advantages can also be observed in patients with an LVEF ranging from 40 to 49%. Contrarily, those benefits cannot be attributed to patients with concomitant AF and HF, regardless of the LVEF value, nor can they be applied to individuals with LVEF ≥ 50% in sinus rhythm.

Although a lower HR is generally beneficial for patients with cardiovascular disease and HF, current data suggests this is not the case in the setting of HFpEF. Per existing guidelines, beta-blockers are not indicated as a primary treatment for HFpEF [24, 25].

This review aims to assess current data regarding the impact of anti-bradycardia pacing in patients with HFpEF.

## Materials and methods

### Search strategy

A search was conducted on databases such as PubMed, ScienceDirect, Springer, and Wiley Online Library using the keywords: HFpEF, diastolic heart failure, left ventricular diastolic dysfunction, cardiac pacing, heart rate, pacemaker, cardiac resynchronization therapy, chronotropic incompetence, exercise intolerance, heart failure with normal ejection fraction, optimization of rate adaptation, and rate-adaptive pacing. The databases were last searched in July 2023.

Articles were published in the following papers: The American Journal of Cardiology, Journal of the American College of Cardiology, Heart Failure, EP Europace, European Journal of Heart Failure, Cardiovascular Drugs and Therapy, American Heart Journal, European Heart Journal, European Journal of Heart Failure, Jama Cardiology, and Pacing and Clinical Electrophysiology.

All five investigators used Google Sheets to summarize the data collected from each article and decide if those studies respected the inclusion and exclusion criteria. Microsoft Excel was used to exclude duplicate articles and to create the tables presented.

### Inclusion and exclusion criteria

**Fig. 2:**
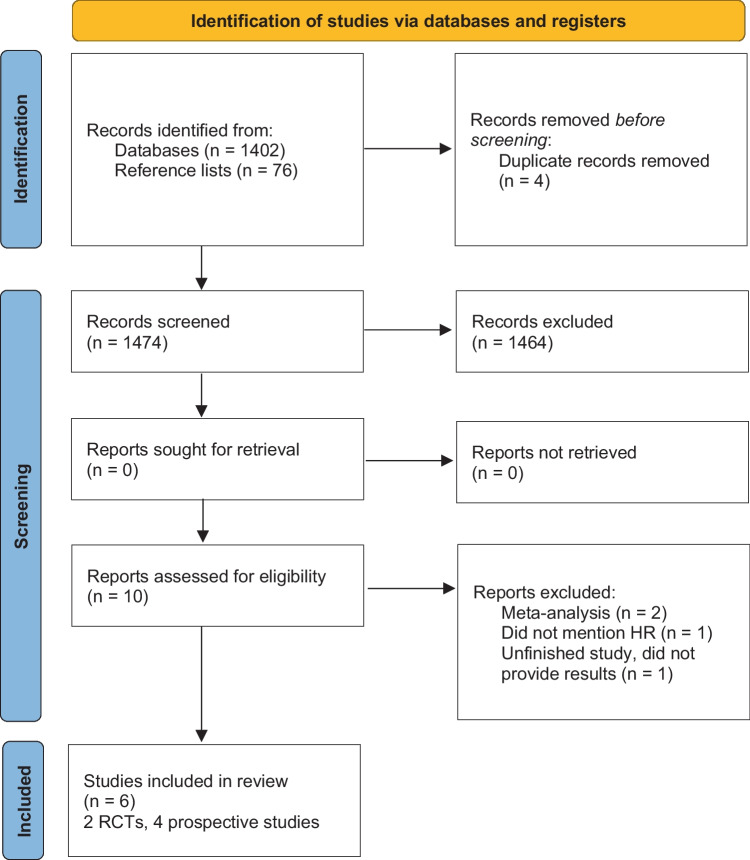
PRISMA study selection flow diagram. Graphics program used: Microsoft Corporation. (2018). Microsoft Word. Retrieved from https://office.microsoft.com/word

Titles and abstracts were screened and analyzed. The full text was reviewed when the articles did not provide enough information in the title and abstract. Inclusion criteria were studies that assessed patients with HFpEF and cardiac pacing therapy who were under optimal pharmacological therapy and had prior or de novo pacemaker implantation. Systematic reviews, case reports, studies with less than six patients or follow-ups of less than one month, and studies that did not include HR were excluded. Ten reports were assessed for eligibility, further excluding four more. See Fig. 2 for the PRISMA study selection flow diagram.

### Data extraction

Data extraction was performed independently by five reviewers with the primary goal of elaborating and establishing information regarding study characteristics and patient attributes, functional status, and quality of life (QoL).

Extracted data regarding study characteristics involved title, authors, year of publication, study design, follow-up time, sample size, presence/absence of pacemaker, and set HR.

Patient attributes included age, sex, HR, comorbidities, medication, echocardiographic measures, and electrocardiogram (ECG) characteristics. Variables extracted to assess HFpEF included NYHA staging, LVEF, NT-proBNP, evidence of preserved LV systolic function, and diastolic dysfunction.

Functional status was assessed using the 6-min walk test (6MWT), pacemaker-detected activity levels, NYHA class, NT-proBNP, peak aerobic capacity (peak *V̇*_O2_), ventilatory efficiency (*V̇*_*E*_/*V̇*_CO2_), *V̇*_O2_ at anaerobic threshold (*V̇*_O2, AT_), and peak exercise stroke volume. Data regarding quality of life included the Minnesota Living with Heart Failure Questionnaire (MLHFQ) and Kansas City Cardiomyopathy Questionnaire Overall Summary Score (KCCQ-OSS). Objective evidence of cardiac structural and functional abnormalities consistent with the presence of LV diastolic dysfunction/raised LV filling pressures are the following echocardiographic parameters: left atrium (LA) volume index > 34 ml/m^2^ (sinus rhythm), *E*/*e*′ ratio at rest > 9, and sPAP > 35 mmHg [24]. Echocardiographic parameters such as LVEF, LA volume, *E*/*A* ratio, *E*/*e* ratio, and systolic pulmonary arterial pressure (sPAP) were extracted from the articles selected.

### Risk of bias

The risk of bias was assessed to evaluate the methodological quality and potential sources of bias of the included studies, as recommended by the PRISMA guidelines. The included articles had a variable follow-up period between 4 weeks and one year. Furthermore, the reason the pacemaker was implanted in HFpEF patients was not assessed, and the higher preset HR was different in the studies analyzed.

Adequate blinding of study participants and investigators is difficult, if not impossible, to accomplish when the HR is changed [13]. Some patients presented with complications or symptoms during pacing, such as palpitations or chest discomfort [26].

One pilot study [27] with six patients had highly selective criteria; therefore, their results can be applied only to a specific type of patient: severe HFpEF with interatrial conduction delay, short left atrioventricular interval during electrophysiological studies, a restrictive filling pattern and no standard indication for a pacemaker.

## Results

Out of ten studies first selected, only six met the inclusion and exclusion criteria. Four were prospective studies with 90 patients analyzed (atrial pacing 26.66%, mixed atrial/ventricular pacing, or ventricular pacing 73.34%). Two were randomized controlled trials with a total of 129 patients assessed (atrial pacing 54.26%, mixed atrial/ventricular pacing, or ventricular pacing 45.73%) (Table 1).

**Table 1:** Studies included in this review, having met the inclusion and exclusion criteria

No. Crt	Title	Type of study	No. of patients	Follow- up	Pacing type*	Study design
1	Effects of a Higher Heart Rate on Quality of Life and Functional Capacity in Patients With Left Ventricular Diastolic Dysfunction [13]	Prospective study	20	6 weeks	***Atrial***: 8/20 ***Ventricular***: • *Standard*:7/20 • *Physiological*: 5/20	A 4-week increase in the lower pacemaker rate to 80 bpm followed by a reversal to the previous lower HR setting from weeks 4 to 6
2	Permanent left atrial pacing therapy may improve symptoms in heart failure patients with preserved ejection fraction and atrial dyssynchrony: a pilot study prior to a national clinical research programme [27]	Pilot study	6	Mean: 25 months	***Atrial***: 6/6 average percentage of atrial pacing for the first 3 months was 96 ± 3%	After 3 months of active pacing, a 2-week randomized double-blind crossover phase compared active vs. inactive LA pacing
3	Safety and Feasibility of a Nocturnal Heart Rate Elevation—Exploration of a Novel Treatment Concept [28]	Prospective	10	6 weeks	***Atrial***: 10/10	A pacemaker-mediated *increase in HR to 100 bpm for 5 h at night* was tested over 4 weeks in 10 patients with diastolic dysfunction
4	A new algorithm for optimization of rate-adaptive pacing improves exercise tolerance in patients with HFpEF [29]	Prospective	54	Mean: 99 ± 19 days	***Ventricular***: • *Standard:* 54/54 VVIR pacing > 90%	A total of 54 patients with HFpEF, permanent AF, and VVIR pacing were randomized to an *intervention group with optimization of rate-adaptation parameters* by using cardiopulmonary exercise testing (CPET) and pacemaker stress echocardiography, and to a *control group with conventional programming*
5	Effect of Personalized Accelerated Pacing on Quality of Life, Physical Activity, and Atrial Fibrillation in Patients With Preclinical and Overt Heart Failure With Preserved Ejection Fraction [10]	RCT	100	Mean: 378 days	***Atrial***: 41/100 ***Mix atrial/ ventricular pacing or ventricular pacing***: 59/100	Randomly assigned to *personalized accelerated pacing* or *usual care* and were followed up for 1 year; the personalized accelerated pacing HR was calculated using a resting HR algorithm based on height and modified by LVEF
6	Rate-Adaptive Atrial Pacing for Heart Failure With Preserved Ejection Fraction The RAPID-HF Randomized Clinical Trial [26]	RCT	29	16 weeks	***Atrial***: 29/29 atrial pacing averaged throughout the pacing phase median, 23.87%	Randomized to *atrial rate-responsive pacing* or *no pacing* first for 4 weeks, followed by a 4-week *washout period* and then *crossover* for an additional 4 weeks

^*^Average percentage of pacing by lead type is not mentioned in all 6 studies

### Non-RCT prospective studies

#### NYHA class

The evolution of NYHA classes has been documented in one study [27], showing improvement at follow-up. In the pilot study, the baseline NYHA class was ≥ III, while at the 3-month follow-up, all patients had NYHA < II [27].

#### NT-proBNP

There is a discrepancy between the findings presented by the analyzed prospective studies. Wahlberg et al. [13] outlined a trend towards a decrease in NT-proBNP when increasing the lower rate setting of pacemakers to 80 bpm in patients with diastolic dysfunction: Visit A (baseline) 915 ± 920 pg/dl compared to Visit B (80 bpm) 815 ± 731 pg/dl, absolute change in NT-proBNP of 100 ± 452 pg/dl (*p* = 0.35), whereas Laurent et al. [27] assessed patients that had a baseline BNP higher than average, presenting an impressive decrease in patients that underwent permanent LA pacing therapy: from 5700 ± 2000 to 2680 ± 1200 pg/ml (*p* = 0.02). In one prospective study, NT-proBNP and troponin did not significantly change at 3-month follow-up in the personalized accelerated pacing group [28].

#### 6MWT

6MWT improved after the patients had been paced at 80 bpm for four weeks compared with baseline, from 329 ± 116 to 350 ± 127 m (*p* = 0.05) [13]. Patients after three months of permanent LA pacing therapy had an increase in the mean distance: 21% greater, 240 ± 25 m vs. 190 ± 15 m (*p* < 0.05) [27]. Values also improved in the intervention (personalized accelerated pacing) group from baseline, 351 ± 72 to 423 ± 53 m at 3-month follow-up compared to a control group from baseline, 357 ± 87 to 374 ± 97 m at follow-up [29]. 6MWT distance also increased by an average of 23 ± 19%, from 258 ± 55 to 322 ± 99 m (*p* = 0.01) [28].

#### MLHFQ

The score decreased after being paced at 80 bpm (baseline 34 ± 19, four weeks 29 ± 22, *p* = 0.03). After returning the patients to the previous lower HR setting, the MLHFQ scores worsened at six weeks 36 ± 23 [13]. Values also improved in the personalized accelerated pacing group from baseline 36 ± 19 to 27 ± 15 at 3-month follow-up compared to a control group from 33 ± 19 at baseline to 33 ± 24 at follow-up [29].

#### Echocardiography

In the prospective studies that were analyzed, only one study showed a slight improvement in the LVEF from 51 to 53% at 3-month follow-up in the personalized accelerated pacing group [29]. *E*/*A* ratio was also improved in one study from 3.4 ± 1.3 to 1.8 ± 0.9 at 3-month follow-up with *p* < 0.009 [27]. *E*/*e*′ ratio improved from 22.6 ± 4.6 to 15.3 ± 4.3 (*p* < 0.006) [27] and 11.7 ± 3.2 to 10.4 ± 2.9 (*p* = 0.025) [29], both at 3-month follow-up. sPAP decreased from 58.7 ± 7.5 to 45.2 ± 8.8 mmHg [29] and 44 ± 14 to 39 ± 12 mmHg (*p* = 0.001) [27]. One study did not present echocardiographic measurements at follow-up [13], and another did not reveal any significant changes [28].

### Randomized controlled trials

#### my-PACE trial

This study was a prospective, blinded, parallel-group, randomized clinical trial conducted with 107 HFpEF patients with predominantly atrial pacing, conduction system pacing, or biventricular pacing: 50 patients were randomized to personalized accelerated pacing and 57 patients to usual care [10]. Seven patients were lost at follow-up.

For patients requiring ventricular pacing, significant efforts were made to preserve physiological ventricular activation by conduction system pacing or biventricular pacing (Table 2). For patients who did not require ventricular pacing (sinus node disease), a standard right ventricular backup lead was implanted with < 0.1% pacing on follow-up. The resulting QRS complex for ventricular-paced patients was 120 ms in the usual care group and 115 ms in the personalized accelerated pacing.

**Table 2:** Percentage of time pacing in my-PACE trial

Percentage of time pacing by lead type	Usual care (*n* = 52)	Personalized accelerated pacing (*n* = 48)
Atrial pacing, median (IQR) %	57% (13–86)	47% (5–86)
Right ventricular septal pacing, median (IQR) %	0.1% (0.0–1.0)	0.4% (0.0–7.3)
His bundle or left bundle branch, median (IQR) %	96% (37–100)	100% (34–100)
Biventricular pacing, median (IQR) %	100% (100)	100% (100)

As a primary outcome, from baseline to follow-up at one month and one year, MLHFQ scores differed between the two groups: usual care-preliminary from baseline to 1 month, mean MLHFQ score decreased (QoL improved) by 0.9 points, eventually to 1-year mean scores increased by 3.5 points (QoL worsened); personalized accelerated pacing—from baseline to 1 month, mean MLHFQ scores decreased by 10.9 points and decreased by 15.0 points at one year (QoL improved) (*p* < 0.001).

As a secondary outcome, there was a vital relative lowering in NT-proBNP levels (available in 91 patients—50 in usual care, 41 in personalized accelerated pacing group), measured from baseline to 1-month follow-up, in personalized accelerated pacing (mean decrease of 109 pg/dl) compared to usual care (mean increase of 128 pg/dl) (*p* = 0.02). Functional status was compared using pacemaker-detected activity levels, available for 50 patients (24 in usual care, 26 in the personalized accelerated pacing group). From baseline to 1-year follow-up, authors observed an increased mean daily activity by 47 min with personalized accelerated pacing versus a decrease by 22 min with usual care. At one-year follow-up, daily activity levels were higher (median 3.1 h) than in the standard care group (median 2.9 h) (*p* = 0.003).

Considering that this trial included patients with AF, there was observed a reduced relative risk of device-detected AF by 27% in the personalized accelerated pacing group compared to usual care.

#### RAPID-HF trial

This study was a single-center, double-blind, randomized, crossover trial testing the effects of rate-adaptive atrial pacing in patients with symptomatic HFpEF and chronotropic incompetence. A total of 32 patients were recruited; 29 underwent pacemaker implantation and were randomized to atrial rate-responsive pacing or no pacing first for 4 weeks, followed by a 4-week washout period and then crossover for an additional 4 weeks [26]. An increase in HR was significant during submaximal and peak exercise in the pacing-on period (mean, 123 bpm) as compared with the pacing-off period (mean: 109 bpm) (*p* < 0.001).

As a primary outcome, at 4-week follow-up, there was no significant effect of pacing on of *V̇*_O2, AT_ from pacing-off to pacing-on phases (mean difference 0.3 ml/kg/min; *p* = 0.46); also mean *V̇*_O2,AT_ in the pacing off phase was 10.4 ml/kg/min and 10.7 ml/kg/min in the pacing on phase.

No significant difference in the secondary endpoints of peak *V̇*_O2_ (mean difference 0.4 ml/kg/min; *p* = 0.27; mean in the pacing off phase 16.5 ml/kg/min; mean in the pacing on phase 16.8 ml/kg/min) or *V̇*_*E*_/*V*_CO2_ slope (mean difference 0.5; *p* = 0.34; mean in the pacing off phase 34.2; mean in the pacing on phase 34.9) was reported. Furthermore, there was no significant change in NT-proBNP level during pacing-off (-88) compared with pacing-on (− 36) phases (mean difference 53 pg/ml; *p* = 0.53). There was no significant difference in the change in KCCQ-OSS during pacing-off (4.7) compared with pacing-on (3.8) phases (mean difference − 0.9;* p* = 0.86). Cardiac output did not significantly change during pacing-on (10.8 l/min) compared to pacing-off (11.5 l/min) despite the increase in HR (mean difference − 0.7 l/min; *p* = 0.14). Stroke volume significantly decreased at peak exercise during the pacing-on phase (88 ml) compared with the pacing-off (112 ml) phase (mean difference − 24 ml; *p* = 0.02).

To conclude the results of this trial, pacemaker implantation to enhance exercise HR did not improve exercise capacity and was associated with increased adverse events.

All the results are concluded in Table 3 and 4.

**Table 3:** Study outcomes

No	Title	NYHA class	NT-proBNP	6MWT	MLHFQ score	Echocardiography	
1	Effects of a Higher Heart Rate on Quality of Life and Functional Capacity in Patients With Left Ventricular Diastolic Dysfunction [13]		*Baseline:*915 ± 920 pg/dl *80 bpm:* 815 ± 731 pg/dl	*Baseline:* 329 ± 116 m *80 bpm:* 350 ± 127 m ***p* = 0.05**	*Baseline*: 34 ± 19 *80 bpm:* 29 ± 22 ***p* = 0.03** *Reversal:* 36 ± 23		
2	Permanent left atrial pacing therapy may improve symptoms in heart failure patients with preserved ejection fraction and atrial dyssynchrony: a pilot study prior to a national clinical research programme [27]	*Baseline:* NYHA class III for at least 3 months while receiving the optimal medical treatment *3-month follow-up:* NYHA class > II	*Baseline:* 5700 ± 2000 pg/ml *3-month follow-up:* 2680 ± 1200 pg/ml ***p* = 0.02**	*Baseline:* 190 ± 15 m *3-month follow-up:* 240 ± 25 m ***p* < 0.05** 21% improvement at *3-month follow-up*		***E*/*A*** **ratio** *Baseline*: 3.4 ± 1.3 *3-month follow-up:* 1.8 ± 0.9 ***p* < 0.009** ***E*/*e*′ ratio** *Baseline:* 22.6 ± 4.6 *3-month follow-up:* 15.3 ± 4.3 ***p* < 0.006** **sPAP** *Baseline:* 58.7 ± 7.5 mmHg *3-month follow-up:* 45.2 ± 8.8 mmHg	
3	Safety and Feasibility of a Nocturnal Heart Rate Elevation—Exploration of a Novel Treatment Concept [28]	–	NT-proBNP and troponin *did not* *significantly* change with the intervention	*Baseline*: 258 ± 55 m *Post-* *intervention:* 322 ± 99 m ***p* = 0.01**		did not reveal any changes in LVEF	
4	A new algorithm for optimization of rate-adaptive pacing improves exercise tolerance in patients with HFpEF [29]			Intervention group *Baseline*: 351 ± 72 m *3 months*: 423 ± 53 m Control group *Baseline*: 357 ± 87 m *3 months*: 374 ± 97 m	Intervention group *Baseline*: 36 ± 19 *3 months*: 27 ± 15 Control group *Baseline:* 33 ± 19 *3 months*: 33 ± 24	Intervention group **LVEF** *Baseline*: 51% *3 months*: 53% **LA volume** *Baseline*: 108 ml *3 months*: 95 ml ***p* = 0.026** ***E*/*e*′** *Baseline*: 11.7 ± 3.2 *3 months*: 10.4 ± 2.9 ***p* = 0.025** **sPAP** *Baseline*: 44 ± 14 mmHg *3 months*: 39 ± 12 mmHg ***p* = 0.001**	Control group **LVEF** 53% 55% **LA volume** 101 ml 103 ml ***p* = 0.026** ***E*/*e*′** 11.9 ± 3 11.8 ± 4.7 ***p* = 0.025** **sPAP** 37 ± 8 mmHg 38 ± 8 mmHg ***p* = 0.001**
5	Effect of Personalized Accelerated Pacing on Quality of Life, Physical Activity, and Atrial Fibrillation in Patients With Preclinical and Overt Heart Failure With Preserved Ejection Fraction [10]		1-year of follow-up: *personalized accelerated pacing*: mean decrease of 109 pg/dl **vs** *usual care:* an increase of 128 pg/dl ***p* = 0.021**		*Personalized accelerated pacing*: **baseline**, 26 (8–45); at 1 month, 15 (2-25); at 1 year, 9 (4-21); ***p* < 0.001** *Usual care*: **baseline**, 19 (6–42); **at 1 month**, 23 (5–39); **at 1 year**, 27 (7–52); ***p* = 0.03**

**Table 4:** Results of RAPID-HF randomized clinical trial

No	Title	NT-proBNP	*V̇*_O2, AT_	Peak *V̇*_O2_	*V̇*_*E*_/*V̇*_CO2_ slop	Change in KCCQ-OSS
1	Rate-Adaptive Atrial Pacing for Heart Failure With Preserved Ejection Fraction The RAPID-HF Randomized Clinical Trial [26]	Did *not* reveal any mean change from the beginning of the pacing-off/pacing-on phase to the end of that 4-week phase *Pacing-off*: − 88 pg/ml *Pacing-on*: − 36 pg/ml ***p* = 0.53**	*Pacing-on*: 10.7 ml/kg/min *Pacing-off*: 10.4 ml/kg/min ***p* = 0.46**	*Pacing-on*: 16.8 ml/kg/min *Pacing-off*: 16.5 ml/kg/m	*Pacing-on:* 34.9 *Pacing-off:* 34.2 ***p* = 0.34**	*Pacing-on:* 3.8 *Pacing-off:* 4.7 ***p* = 0.86**

## Discussion

We conducted a review to investigate how changing the baseline heart rate (HR) preset in patients with heart failure with preserved ejection fraction (HFpEF) and cardiac pacing therapy affects outcomes. Our findings were as follows:

- A temporary increase in HR was not associated with worse outcomes.
- An average 24-h increase in HR, rather than an enhanced exercise HR, improves the quality of life and potentially outcomes in patients with HFpEF.
- The lead location and pacing mode may influence these benefits:
  - For atrial pacing, Bachman bundle pacing may influence outcomes in specific subgroups of HFpEF patients, compared to other types of right atrial pacing.
  - For ventricular pacing, physiological pacing (conduction system pacing or biventricular pacing) may be necessary to obtain the above-mentioned clinical benefits.

Based on our review, we found that a higher resting heart rate by atrial pacing has been proven to be beneficial in HFpEF. However, it was observed that a higher adaptive rate during exertion by atrial pacing did not have the same positive effect. Overall, the studies included in this review suggest that higher heart rates (especially atrial rates) through cardiac pacing may improve symptoms and potentially lead to better outcomes in HFpEF.

**Fig. 3:**
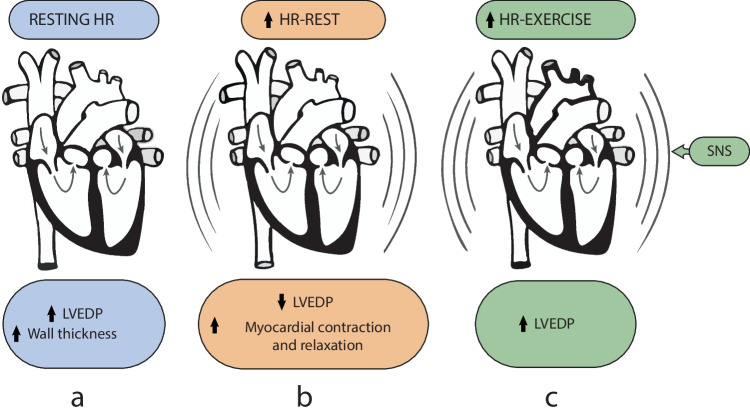
Influence of the HR on pathophysiology of HFpEF patients a. During rest HR, LVEDP, and wall thickness are increased at any volume due to passive stiffness. b. Increased HR during rest, using a pacemaker, LVEDP decreases due to enhanced myocardial contraction and relaxation. c. During exercise, LVEDP increases because of higher HR along with SNS activation [5, 8, 14–16]. HR, heart rate; LVEDP, left ventricular end-diastolic pressure; SNS, sympathetic nervous system. Graphics program used: Adobe Inc. (2019). Adobe Illustrator. Retrieved from https://adobe.com/products/illustrator

As shown in Fig. 3, the pathophysiology of HFpEF patients is influenced not only by the variability of the HR but also by the context of its increase: a higher HR during rest, using a pacemaker, determines a lower LV filling pressure in contrast to higher HR during exercise, when LVEDP increases. This inconsistency is influenced by the enhancement of another adaptation system—the sympathetic nervous system [5, 8, 14–16].

Researchers found that the impact of beta-blockers in reducing HR is associated with the survival benefit and that an HR of 70 or more bpm is a prognostic risk factor in patients with CAD and LV systolic dysfunction [22, 30, 31]. These findings were used for patients with HFpEF, but the clinical evidence showed that HFpEF and HF with reduced ejection fraction are not similar.

Silverman et al. [32] and Patricia Palau et al. [33] conducted two studies that highlighted an increased risk of mortality associated with beta-blocker usage and the withdrawal of this medication improves maximal functional capacity in patients.

Furthermore, the six studies reviewed showed that maintaining a higher HR has essential benefits in terms of clinical and paraclinical outcomes and, consequently, the usage of beta-blockers provides no improvements, and it should not be considered a standard approach for patients presenting with HFpEF alone. However, in ischemic heart disease or AF with rapid ventricular response in association with HFpEF, beta-blocker therapy should be considered, the focus is on an individualized approach to avoid lowering the HR too much.

Patients with HFpEF have potential risks for harm from greater HR during exercise due to inadequate relaxation and increased LV stiffness, which contribute to increased LV filling pressures, inadequate rate-related increase in systolic performance, and a lower peak exercise HR [14, 26]. Most common repercussions are pulmonary congestion and vascular remodeling, impaired gas exchange and decreased lung diffusion capacity, increased myocardial oxygen demand and wall stress, and potentially worsening ischemia in vulnerable patients. Stiffening of the aorta and abnormal ventricular–arterial interaction can also play a role in the limitation of exercise capacity [14, 34]. Accordingly, HFpEF patients revealed a major increase in proximal arterial stiffness during a moderate level of exercise, which was otherwise absent at rest [35].

When performing a physical activity, healthy individuals are capable of significant increases of cardiac output by about 300% higher than baseline with almost no change in LV filling pressures, whereas HFpEF patients were only able to achieve a 35% increase in cardiac output at the cost of a 50% increase in LV filling pressure [15].

As far as the limitations of this review are concerned, out of the six articles evaluated, one prospective study documented the NYHA class, three studied the MLHFQ results, five studies looked at NT-proBNP, and four studies assessed 6MWT. Only three studies presented echocardiographic parameters (one study *E*/*A* and other the LA volume, two studies the *E*/*e*′, sPAP and LVEF). Furthermore, only one article analyzed peak *V̇*_O2_, *V̇*_*E*_/*V̇*_CO2_, *V̇*_O2,AT_, and peak exercise stroke volume, as well as using KCCQ-OSS.

The review process has encountered limited studies addressing the HR preset in patients with pacemakers and HFpEF. The scarce available studies can restrict the depth and breadth of the review’s analysis and conclusions. Studies on this topic may vary in terms of their design, sample size, patient characteristics, pacemaker technologies, and methodologies used to assess HR presets. The included studies may have variations in patient populations, pacemaker technologies, pacing type, HFpEF diagnostic criteria, study designs, and follow-up durations. The absence of long-term data (Wahlberg et al. 2019) can limit the understanding of sustained benefits, adverse effects, or changes in clinical outcomes over time.

Future comparative effectiveness trials evaluating different HR thresholds will help refine the current treatment strategy. Therefore, there is a need for new evidence-based guidelines that target HFpEF in particular. Although an individualized approach from the clinician could be considered time-consuming, it will positively impact the patient’s quality of life. Furthermore, the focus should be on evaluating the benefits of different types of pacing: atrial, ventricular, or mixed [36].

Our review underlines the limited data currently available on anti-bradycardia pacing therapy in HFpEF patients, with a limited number of trials, all using different protocols, including a relatively small population of patients with different underlying heart and electrical diseases and short-term follow-up.

## Conclusion

The widespread recognition of HFpEF as a clinical entity and, therefore, the increased number of diagnoses of HFpEF requires a more individualized approach and quality of life management. Our review demonstrates that higher resting HR by atrial pacing may improve symptoms and even outcomes in HFpEF, while a higher adaptive rate during exertion has not been proven beneficial.

## Data Availability

Not applicable.
